# Trauma-toxicology: concepts, causes, complications

**DOI:** 10.1007/s00210-023-02845-3

**Published:** 2023-11-24

**Authors:** Holger Barth, Franz Worek, Dirk Steinritz, Panagiotis Papatheodorou, Markus Huber-Lang

**Affiliations:** 1 Institute of Experimental and Clinical Pharmacology, Toxicology and Pharmacology of Natural Products, University of Ulm Medical Center, Ulm, Germany; 2 Bundeswehr Institute of Pharmacology and Toxicology, Munich, Germany; 3 Institute of Clinical and Experimental Trauma Immunology, University of Ulm Medical Center, Ulm, Germany

**Keywords:** Trauma, Toxicology, Chemical warfare, Biological warfare, Bacterial toxins, Barrier damage, Post-traumatic complications, *Clostridioides difficile*, Pharmacologic options

## Abstract

Trauma and toxic substances are connected in several aspects. On the one hand, toxic substances can be the reason for traumatic injuries in the context of accidental or violent and criminal circumstances. Examples for the first scenario is the release of toxic gases, chemicals, and particles during house fires, and for the second scenario, the use of chemical or biological weapons in the context of terroristic activities. Toxic substances can cause or enhance severe, life-threatening trauma, as described in this review for various chemical warfare, by inducing a tissue trauma accompanied by break down of important barriers in the body, such as the blood-air or the blood-gut barriers. This in turn initiates a “vicious circle” as the contribution of inflammatory responses to the traumatic damage enhances the macro- and micro-barrier breakdown and often results in fatal outcome. The development of sophisticated methods for detection and identification of toxic substances as well as the special treatment of the intoxicated trauma patient is summarized in this review. Moreover, some highly toxic substances, such as the protein toxins from the pathogenic bacterium *Clostridioides* (*C*.) *difficile*, cause severe post-traumatic complications which significantly worsens the outcome of hospitalized patients, in particular in multiply injured trauma patients. Therefore, novel pharmacological options for the treatment of such patients are necessarily needed and one promising strategy might be the neutralization of the toxins that cause the disease. This review summarizes recent findings on the molecular and cellular mechanisms of toxic chemicals and bacterial toxins that contribute to barrier breakdown in the human body as wells pharmacological options for treatment, in particular in the context of intoxicated trauma patients. “trauma-toxicology” comprises concepts regrading basic research, development of novel pharmacological/therapeutic options and clinical aspects in the complex interplay and “vicious circle” of severe tissue trauma, barrier breakdown, pathogen and toxin exposure, tissue damage, and subsequent clinical complications.

## Introduction

**Fig. 1:**
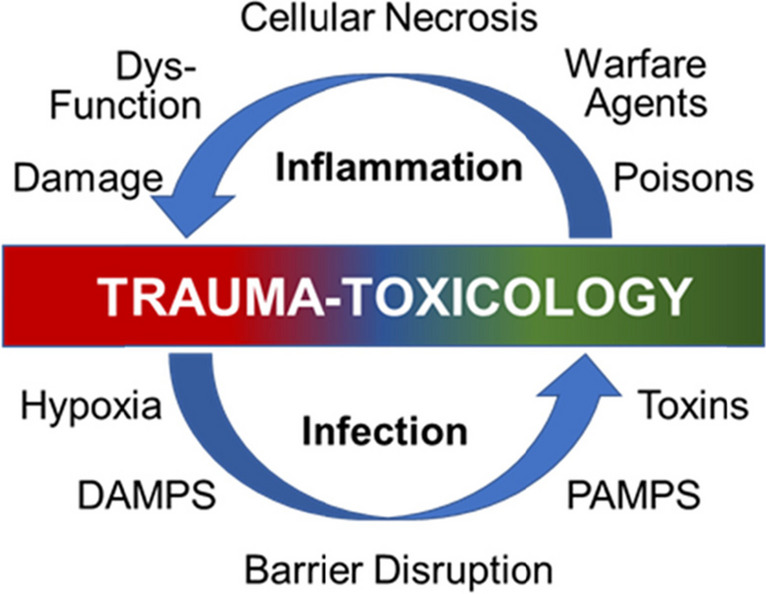
The concept of trauma-toxicology: The interplay between trauma and toxic agents and their biological consequences. Explanations are given in the text

Physical trauma is the realization of an external danger impact which threatens any life at any time. In humans and animals, the physical trauma force vector can breach the protective layers encompassing of the skin, fasciae, capsules, and underlying tissues. This damage results in the generation and release of damage-associated molecular patterns (DAMPs) including membrane debris, mitochondrial components, histones, DNA- and RNA fragments, and damaged proteins (Huber-Lang et al. 2018) (see Fig. 1). The demolished and compromised barriers inadequately restrict the efflux of vital internal constituents, such as blood, into the external environment, leading to circulatory disturbances, hypoperfusion, hypoxia, and shock. Conversely, these compromised external and internal barriers become highly susceptible to the ingress of microorganisms and potentially hazardous substances and fluids (Huber-Lang et al. 2018), thus increasing the risk of infectious, toxic, or septic complications. Recent findings from our laboratory have demonstrated that a highly standardized murine polytrauma plus hemorrhagic shock results in remote intestinal injury characterized by an enhanced permeability of the gut-blood-barrier (GBB) (Wrba et al. 2019), which can ultimately impair organ performance and culminate in the development of multiple organ dysfunction syndrome (MODS), often resulting in a fatal outcome (Huber-Lang et al. 2018). An effective yet limited sealing system of the disrupted tissues is the fluid-phase and cellular coagulation system, which rapidly activates in the aftermath of trauma to prevent leakage (Rossaint et al. 2023). Similarly, activation of the interconnected innate complement cascade (Burk et al. 2012) and the “first cellular line of defense” strive to repel microbial invasion and to inhibit their growth.

Trauma as a physical “wound” can manifest across various contexts, including civilian incidents, acts of terrorism or within military operations. It encompasses physical injuries, burns, as well as exposure to chemical, biological, or radiological/nuclear (CBRN) warfare agents. While bioterrorism and B-warfare—so far known—predominantly involve agents such as viruses, bacteria, and bacterial toxins (Botulinum Neurotoxin (Kreyden et al. 2000), Anthrax toxin) as well as plant toxins (Ricin) (Tin et al. 2022), the arsenal of chemical warfare agents is extensive (Wille et al. 2011) and comprises toxic substances that affect the skin, the lungs, or the nervous system. The latter comprises organophosphates such as tabun, sarin, VX, and the Novichok compounds, which can cause a severe, life-threatening cholinergic syndrome, as described in more detail later. These agents possess nearly boundless potential for inflicting tissue damage.

Furthermore, combinations of traumata in the spatio-temporal dimension, i.e., simultaneously across different body regions or consecutive impacts, can aggravate the overall trauma load. As a side note, the concept of trauma extends beyond physical injuries to encompass psychosocial dimensions. Research has unveiled a growing understanding of the intricate physio-psychological interactions associated with trauma (Haffner-Luntzer et al. 2019). Notably, psychological stress has been shown to significantly alter the composition of the gut microbiome, potentially influencing the presence of exo- and endotoxin-producing microbes (Langgartner et al. 2018). However, the focus of this review does not encompass psychological aspects of trauma, which remain beyond the scope of our discussion.

## Chemical warfare agents

Chemical warfare represents a dark chapter in the history of armed conflicts, encompassing the deliberate use of chemical substances to inflict harm upon adversaries. Unlike conventional weapons, which rely on explosives or projectiles, chemical weapons leverage the toxic properties of chemicals to cause injury, incapacitation, or death. Natural toxins from plants or animals can be regarded as the earliest types of chemical warfare agents (CWAs). One well-known example is curare, a poison derived from certain plants found in South America that works by blocking neuromuscular transmission, leading to muscle paralysis and ultimately respiratory failure (Bowman 2006).

World War I (WWI, 1914 to 1918) stands as a pivotal moment in the history of warfare: The first large-scale use of chlorine gas from pressurized canisters across a 5-mi front by the German forces, engulfing Allied positions, has to be regarded as “zero hour” of chemical warfare in modern history (Black 2016). During WWI, the deployment of CWAs in massive quantities (about 125,000 tons), resulted in about 90,000 fatalities and 1.3 million nonfatal casualties. The use, large-scale development, production, stockpiling, or transfer of chemical weapons or their precursors is meanwhile prohibited by the Chemical Weapons Convention that entered into force in 1997. Nonetheless, chemical weapons have been utilized in a number of subsequent conflicts, terrorist attacks, or assassinations (Ganesan et al. 2010). The most recent incidents include the murder of Kim Jong-nam (VX) and the attempted killings of Sergei Skripal and Alexei Navalny (both with Novichok) (Brunka et al. 2022).

### Classes of chemical agents

Chemical warfare agents are categorized into several groups based on their chemical properties, mechanisms of action, and pathophysiological effects. The most common classification system divides them into six categories (see Table 1).

**Table 1:** Main classes of chemical agents and their mode of action

Classes of chemical agents	Mode of action
Blister agents (or Vesicants) (e.g., sulfur mustard)	Alkylation of biomacromolecules (e.g., DNA, proteins)
Chemical asphyxiants (“blood agents”) (e.g., cyanide)	Inhibition of the respiratory chain by inhibition of mitochondrial complex IV
Pulmonary agents (e.g., phosgene or chlorine)	Bi-phasic: 1. Irritation of mucous membranes/peripheral nerve endings 2. Development of lung edema and toxic lung injury
Nerve agents (e.g., sarin, VX, Novichok)	Inhibition of acetylcholinesterase (AChE)
Riot control agents (e.g., tear gases) and incapacitating agents (e.g., adamsite)	Interaction with peripheral sensory nerve endings
Mental incapacitating agents (“pharmacological-based agents”) (e.g., fentanyl)	Interaction with neuronal targets expressed in the central and peripheral nervous system (e.g., opioid receptors)

Blister agents cause severe skin, eye, and mucous membrane damage. They are named for the large, painful blisters that they can cause. Blood agents are compounds that interfere with the body’s ability to utilize oxygen. They are typically absorbed through the respiratory system but can also be swallowed. Examples include hydrogen cyanide (AC) and cyanogen chloride (CK). Pulmonary agents damage lung tissue, often leading to suffocation. Examples include chlorine (Cl), phosgene (CG), and chloropicrin (PS). Nerve agents disrupt the neuronal signal transmission, leading to cholinergic overstimulation. Examples include sarin (GB), soman (GD), tabun (GA), VX, and the Novichoks.

In addition to these, there are also riot control agents, which are chemicals used for law enforcement and crowd control. These chemicals, while not intended for lethal use, can cause immediate irritation of the eyes, nose, and respiratory tract. Mental incapacitating agents include drugs and compounds that affect functions of the central nervous system resulting in respiratory depression and loss of consciousness. Examples are fentanyl and derivatives thereof. Because these compounds are used as drugs in clinical routine, they are also referred to as “pharmacological-based agents.” Furthermore, some chemical agents may exhibit characteristics of multiple categories. For example, certain compounds can act as both blister agents and pulmonary agents, depending on the route of exposure.

Chemical warfare compounds have the potential to impair the exposed person’s health instantly or within a few hours after exposure. Trauma damage sustained during combat or due to the explosive deployment of the warfare agent may exacerbate the health condition. As a result, the intoxicated trauma patient requires both trauma care and additional treatment due to chemical agent contamination.

### The intoxicated trauma patient

The management of this specific group of patients is difficult, yet it adheres to strict guidelines (Wille et al. 2019). First, early antidote treatment (e.g., atropine and obidoxime in the event of nerve agent poisoning (Amend et al. 2020) or naloxone in the case of opioid is life-saving and should be initiated as soon as possible. The “time-until-first-treatment” is critical and should be kept as short as feasible. Antidotes can be delivered using auto-injectors while still in the hot zone and prior to decontamination. The “trigger-to-treat” is usually the manifestation of characteristic chemical agent-related clinical symptoms. However, particularly in the event of percutaneous exposure to non-volatile nerve agents (i.e., VX or Novichoks), symptoms may not manifest immediately, despite the fact that the patient has already been severely poisoned.

Onsite testing of acetylcholinesterase activity using a mobile test kit and the use of a sensitive and easy to use organophosphate (OP) skin disclosure kit may allow for the early diagnosis of OP skin exposure and the start of life-saving countermeasures (Worek et al. 2016). Contaminated patients cannot be sent into the rescue chain without prior decontamination. Massive bleedings and trauma injuries must also be handled prior to decontamination, for example, using tourniquets, to minimize any treatment delays. In a mass intoxication scenario, the number of patients will exceed the available resources (Rossaint et al. 2023). Thus, there is an urgent need to prioritize equipment and medical supplies using triage algorithms (Khoshnevis et al. 2015). It is critical to repeat the triage procedure on a regular basis in order to detect deteriorating health issues, particularly following percutaneous exposures.

### Verification of exposures to chemical warfare agents

Most chemical agents are highly reactive. They readily hydrolyze following either an enzymatic or, more typically, a non-enzymatic pathway. Moreover, they can form covalent products (adducts) with endogenous biomacromolecules (e.g., DNA and proteins) (John & Thiermann 2021). The limited stability and high reactivity of most chemical agents preclude the detection of the intact poison in vivo, thus requiring the search for more stable and long-lived surrogate parameters derived from biotransformation (John et al. 2018). Thus, detection of the metabolites or the adducts can be used to verify human exposures. Gas and liquid chromatography (LC) coupled to mass spectrometry (MS) are commonly used for biomedical verification of OPNA (organophosphorus nerve agent) exposure in humans and animals (Kranawetvogl et al. 2023).

## Barrier failure due to trauma

**Fig. 2:**
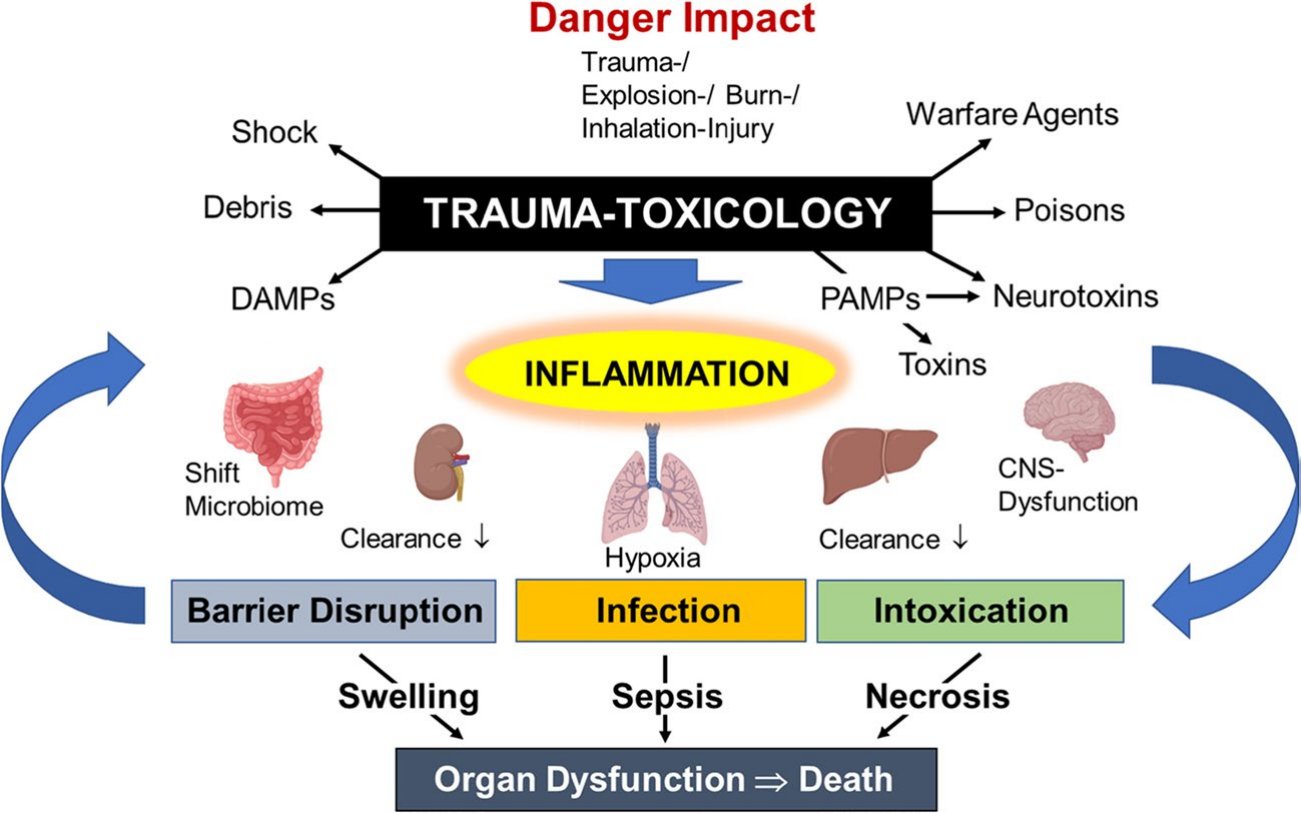
The multiple aspects of trauma-toxicology. Explanations re given in the text. CNS, central nerve system; DAMPs, danger-associated molecular pattern; PAMPs, pathogen-associated molecular pattern

An intact intestinal barrier serves as a crucial boundary separating the systemic circulation from the intestinal microbiome. When this barrier is compromised, it can lead to the translocation of bacteria and pathogen-associated molecular patterns (PAMPs) into the bloodstream or lymphatic system, thereby intensifying the immune response and promoting systemic inflammation (Wrba et al. 2017). This phenomenon is commonly observed as a complication following injury, with profound systemic effects on both pro-inflammatory and anti-inflammatory immune responses, as well as organ perfusion and oxygenation. Such intestinal barrier dysfunction often contributes to multiple organ dysfunction in the clinical course post-injury (Faries et al. 1998; Spindler-Vesel et al. 2006). In case of direct abdominal trauma, experimental evidence suggests, that the infliction of macroscopically visible intestinal injuries is significantly influenced by the location and intensity of the traumatic force (Maitz et al. 2021). In the clinical setting, indirect, remote abdominal traumata appear more frequent than direct ones. Especially, severe blood loss with development of hemorrhagic shock during or after traumatic injury emerges as a major factor causing endothelial damage and subsequent dysfunction of the intestinal barrier (see Fig. 2).

Our own research findings further emphasize the vulnerability of the intestines to hypoperfusion due to blood loss or centralization. In patients with multiple injuries, we observed a remarkable increase in the levels of circulating glycocalyx components and markers of intestinal injury and permeability, particularly in those who had experienced substantial blood loss (Halbgebauer et al. 2018). In a murine model of multiple trauma and hemorrhage, we observed several notable effects on intestinal integrity even in the absence of direct abdominal injury. These effects included an increase in abdominal girth, indicative of extravascular fluid accumulation, a reduction in the expression of the central tight-junction protein, zonula occludens protein 1, in cell–cell contacts within the ileum and colon, and the appearance of mucosal molecules in the bloodstream (Wrba et al. 2019).

These findings underscore the significant impact of systemic post-traumatic processes on the integrity of the intestines. Additionally, it is worth noting that traumatic brain injury, as demonstrated in various preclinical models, can additionally impair intestinal barrier function (Bansal et al. 2010; Feighery et al. 2008; Ma et al. 2019). Mechanistically, we have identified the local upregulation of an apoptosis-inducing protein, thirty-eight-negative kinase 1, as a potential mediator of post-traumatic intestinal epithelial cell death (Armacki et al. 2018). For future therapeutic applications, preventing the effects of mesenteric “lymph toxicity” (Magnotti et al. 1998; Deitch et al. 2006; Fang et al. 2010; Levy et al. 2013) may aid in reducing the detrimental effects of intestinal injury on remote organ systems. Taken together, the available data underscores the significance of the intestine as an often-underappreciated contributor to the development of post-traumatic complications.

## Trauma-caused microbiome shifts: role of bacterial enterotoxins

The human as “macrocosm” and the integrated and surrounding microorganisms as “microcosm” share a complex and mutually dependent relationship. In the experimental setting of rodent polytrauma, both, others, and our research efforts have uncovered rapid microbial alterations within the gut mere hours after injury, a phenomenon influenced, among other factors, by the systemic inflammatory response and the demand for catecholamines (Nicholson et al. 2018; Appiah et al. 2021). In the days following trauma, a significant shift in the microbiome is observed, characterized by a transition towards a pathobiome. This shift manifests as a loss of beta-diversity and the prevalence of certain microorganisms such as *Rothia*, *Anaerostipes*, and *Lactobacillus*. Simultaneously, indications of a compromised intestinal barrier emerge, as recently demonstrated in a rat model of multiple injuries and secondary insults (Munley et al. 2023). Studies employing rodent burn injury revealed that advanced age exacerbates microbiome dysbiosis and weakens the host’s antimicrobial defenses (Wheatley et al. 2020). Conversely, adolescent rats subjected to repetitive mild traumatic brain injury (TBI), preceded by microbiome depletion, exhibited a pathogenic state dominated by *Clostridia*, rendering them more vulnerable than their adult counterparts (Sgro et al. 2022).

Translational research has consistently revealed that microbial changes are closely associated with adverse outcomes (Schuijt et al. 2013). Several factors, notably trauma-induced hypoxia, stress, and administration of antibacterial drugs, have been identified as key drivers of microbiome alterations. In patients with persistent hypoxia (paO_2_/FiO_2_ ratio below 300) following burn and inhalation injuries, an altered bronchoalveolar microbiome is evident, characterized by an enrichment of *Prevotella*, *Corynebacteria*, and *Mogibacterium* (Walsh et al. 2017). Likewise, spinal cord injury studies, both experimental and clinical, have identified an increase in inflammation-promoting microbes, including *Clostridia* (Valido et al. 2022). Despite the growing body of evidence pointing to early post-trauma microbiome alterations, our understanding of the specific pathobiome and the mechanisms involved in rebalancing microbiome homeostasis following trauma remains limited. Further investigations are warranted to unravel these intricate interactions.

Numerous microorganisms possess the capability to facilitate microbial invasion by strategically targeting and compromising the intestinal barrier through the action of enterotoxins. These enterotoxins act predominantly in the gut and mainly target the intestinal epithelial cells, often exhibiting pore-forming properties, or disrupt the integrity of intercellular tight junctions, resulting in a loss of barrier function. Enterotoxins can be produced by a variety of pathogens, including *Staphylococcus aureus*, *Bacillus cereus*, and *Streptococcus pyogenes*, and by plants (Ricin), as summarized in Table 2.

**Table 2:** Main toxins involved in traumatic injuries and post-traumatic complications

Bacterium (Plant)	Toxin	Mechanism of action (molecular, cellular)	Clinical consequences, disease
*C. difficile*	TcdA/TcdB CDT	Enterotoxins, Glucosylation of Rho ADP-ribosylation of actin Cytoskeleton breakdown, cell rounding	Loss of barrier function in gut, diarrhea, recruitment of immune cells, inflammation, pseudomembranous colitis (post-traumatic complications)
*Bacillus anthracis*	LT (lethal toxin) ET (edema toxin)	Cleavage of MAP kinases, macrophage killing cAMP increase, edema	Dependent on uptake route; gastrointestinal anthrax; inhalational anthrax: most severe, often fatal (B-warfare)
*Clostridium botulinum*	BoNT (neurotoxin)	Cleavage of synaptobrevin/VAMP proteins in motoneurons, exocytosis of ACh inhibited	Flaccid paralysis, death; botulism (B-warfare)
*Staphylococcus aureus* (Pinchuk et al. 2019; Kaempfer et al. 2002)	SEA (enterotoxin A/B) SEB (enterotoxin B) TSST-1 (toxic shock syndrome toxin-1)	Superantigens, bind to class II MHC molecules on antigen presenting cells, massive T cell activation, massive cytokine release Superantigen, activates T-lymphocytes, excessive cytokine production	Excessive cellular immune response, toxic shock, SEA: food poisoning (SEB considered as B-warfare) High fever, vomiting, diarrhea, low blood pressure, seizures, etc
*Bacillus cereus*	NHE (nonhemolytic enterotoxin)	Activates nod-like receptor protein-3 (NLRP3) inflammasome, pyroptosis, apoptosis, inflammation of infected tissue	Food intoxication, gastrointestinal symptoms
*Streptococcus pyogenes* (Kaempfer et al. 2002)		Superantigens, bind to class II MHC molecules on antigen presenting cells, massive T cell activation, massive cytokine release	Excessive cellular immune response, toxic shock (considered as B-warfare)
*Ricinus communis* (plant: Castor bean)	Ricin	N-glycosidic cleavage of an adenine residue from 28S rRNA, protein synthesis inhibited, cell death	Dependent on uptake route; orally: vomiting, diarrhea (bloody), dehydration, low blood pressure, organ failure, often fatal (B-warfare, bioterroristic agent)

Notably, enterotoxins, especially the AB-toxins produced by *C. difficile* (Aktories 2011) and *Clostridium perfringens*, play a significant role in this context. In serum and wounds of burn and trauma patients, enterotoxin A from *Staphylococcus aureus* (SEA) could be isolated and were predictive of mortality (Ali et al. 2022; Prindeze et al. 2014). In a rat model of infectious burn wounds, enterotoxin B (SEB) and toxic shock syndrome toxin-1 (TSST-1) were found to translocate from the wounds to the kidneys, potentially contributing to the development of remote complications (Mino et al. 2013). Collectively, research on enterotoxins has predominantly centered on burn injuries, necessitating further comprehensive mechanistic investigations in diverse trauma and post-traumatic settings.

## Role of bacterial toxins in traumatic diseases, barrier failure, and post-traumatic complications

Nowadays, due to changing global political situations, there is increasing concern regarding the deployment of C- and B-weapons in the context of military or terroristic activities, also in Europe. Therefore, research on the mode of action of such toxins is of major impact, as well as on the development of novel, highly specific and sensitive detection methods for such compounds and therapeutic options to treat traumatic and post-traumatic diseases caused by C- and B-warfare.

We and others have investigated the mode of action of the protein exotoxins of *C. difficile* on the molecular and cellular levels in detail and suggested a panel of molecules including human body’s own proteins and peptides as well as licensed drugs that are used for therapy of other diseases as potent inhibitors against *C. difficile* toxins. These molecules, in addition to the already available antibacterial drugs against *C. difficile*, might provide an attractive starting point for clinical studies to introduce novel pharmacological options to treat and/or prevent *C. difficile*-induced infections (CDI).

Similar to the trauma response, the response to toxic substances including bacterial protein toxins can result in local and systemic inflammation, activation and depletion of the coagulation- and complement cascade, development of barrier dysfunction, micro- and macro-perfusion problems, and subsequent organ dysfunction and failure (Abrams et al. 2022). Although the clinical consequences of toxins can be similar, toxic compounds and their induced pathophysiology can be rather different. In conclusion, toxic compounds exhibit a wide range of characteristics, yet their clinical manifestations can resemble those of other severe diseases, potentially leading to multiple organ failure. However, little is known about the role of toxins in the context of severe tissue trauma and the potential underlying crosstalk driving the pathophysiology towards disease progress including the process of post-traumatic regeneration. In particular, barrier failure due to toxic compounds such as chemical warfare or toxins (e.g., enterotoxins from *C. difficile*) needs further mechanistic enlightenment.

### Trauma-associated *Clostridioides difficile* infection

In recent decades, there has been a global increase in *Clostridioides* (formerly *Clostridium*) *difficile* infections, impacting not only the traditional demographic of elderly patients with prolonged use of antibacterial drugs (Depestel & Aronoff 2013). In case of accidental or surgical trauma, *C. difficile* infection develop in ca. 1–3% (Gonzalez et al. 2022; Lumpkins et al. 2008). Moreover, in cases of burn injuries, colonization by *C. difficile* on the skin and in the surrounding environment has been reported in up to 18% of cases (Shoaei et al. 2022). An extensive analysis of over 11,000 trauma patients admitted to a level I trauma center revealed a significant association between *C. difficile* infection and a nearly threefold increase in mechanical ventilation requirements, mortality rates, and a markedly prolonged stay in both the intensive care unit (ICU) and overall hospitalization duration (Karamanos et al. 2018).

Patients with post-traumatic *C. difficile* often exhibited a high initial injury severity score (ISS), an abdominal injury pattern (colonic, renal, and hepatic), received third-generation cephalosporins and/or clindamycin, and/or i.v. proton-pump-inhibitors (Karamanos et al. 2018). In addition, a clinical analysis after blunt trauma proposed a specific *C. difficile* patient population that was older than 65 years, and developed greater multiple organ dysfunction scores (including enhanced base deficit, lactic acid, creatinine, glucose levels, and reduced PiO_2_:FiO_2_) than uninfected trauma patients (Vanzant et al. 2015). A recent multifactorial analysis reaffirmed these risk factors and introduced trauma as a surgical cause associated with *C. difficile* infection (Jachowicz et al. 2022).

It is noteworthy that an analysis of approximately 1.5 million surgical patients indicated that an elevated body mass index, a measure of adiposity, appears to confer some protection against *C. difficile* infection (Meier et al. 2019). Recognizing the challenge of *C. difficile* infection in trauma patients, management guidelines have been developed to address this concern (Sartelli et al. 2019, 2021). Therapeutically, application of phosphatidylcholine as a key component of the intestinal mucosal barrier reduced epithelial necrosis and improved the barrier integrity in an in vitro intestinal model of *C. difficile* exposure (Olson et al. 2014). However, translating these findings into clinical interventions for restoring the gut-blood barrier in cases of *C. difficile* infection remains a pending challenge.

### *Clostridioides difficile* toxins as reason for post-traumatic complications

#### Structure, uptake, mode of action, and pathophysiological role of TcdA and TcdB

Toxin A (TcdA) and toxin B (TcdB) are the two major exotoxins of *C. difficile* (Aktories 2011; Just et al. 1995a, b). A third toxin termed CDT (*C. difficile* transferase) is produced by certain epidemic strains (e.g., *C. difficile* strain BI/NAP1/027). All three toxins are protein toxins harboring a deleterious enzymatic domain, which is delivered into target cells via receptor-mediated endocytosis. The toxins’ actions on target tissues are directly responsible for the outcomes or severity of *C. difficile*-associated diseases (CDADs), such as diarrhea and pseudomembranous colitis (Papatheodorou et al. 2018).

**Fig. 3:**
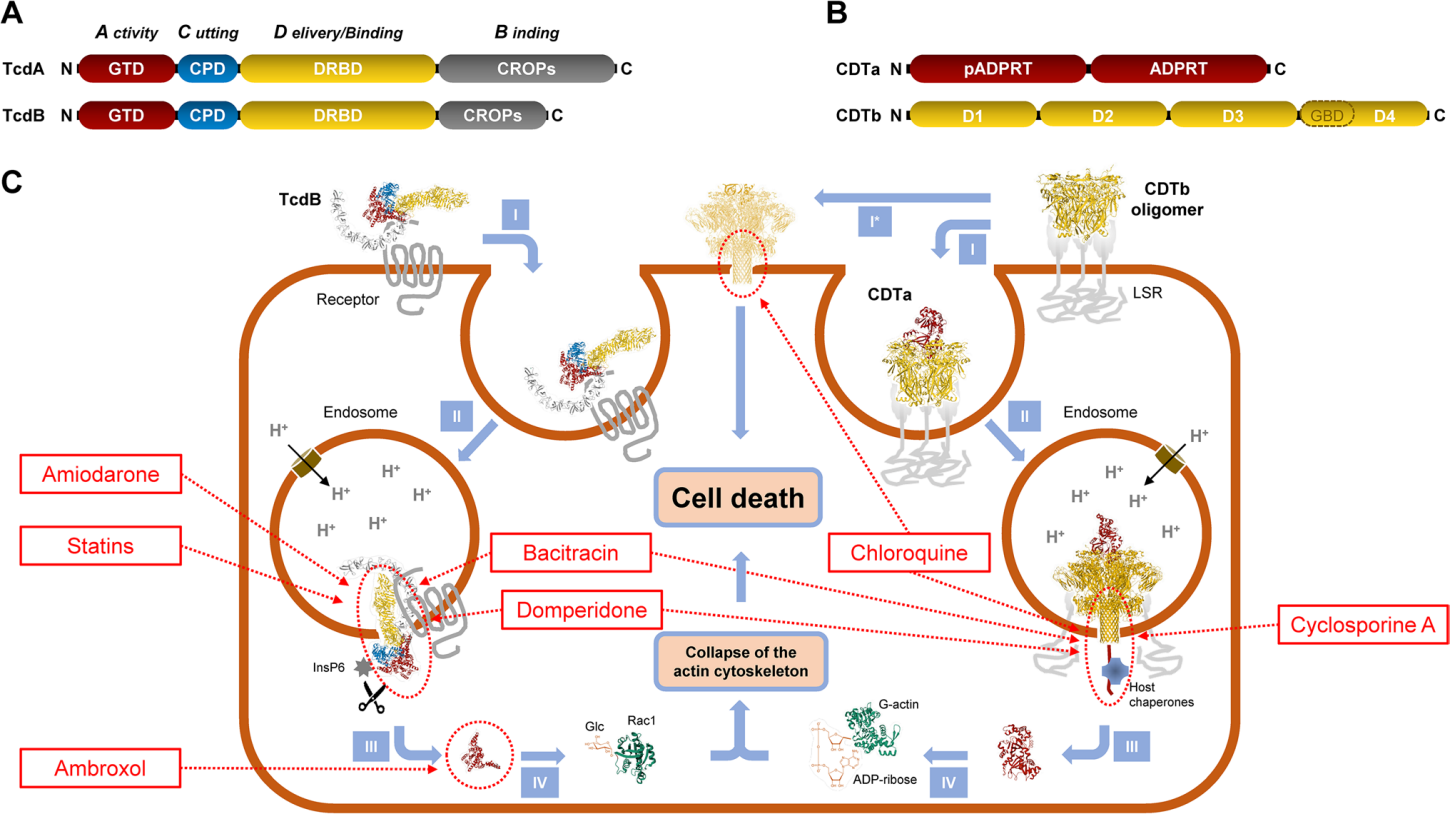
Domain architecture, cellular uptake, mode of action, and inhibitory drugs of *C. difficile* toxins. **A** Domain architecture of TcdA and TcdB. The glucosyltransferase domain (GTD, red) is the toxic part of the toxins that modifies host Rho and/or Ras GTPases upon cleavage and release into the cytosol by the cysteine protease domain (CPD, blue). The combined repetitive oligopeptides (CROPs, gray) act together with the delivery and receptor binding domain (DRBD, yellow) in binding of the toxins to cell surface receptors. The DRBD is also harboring a region that forms a translocation pore in endosomal membranes upon acidification of the endosomes via vacuolar H^+^-ATPases (shown in brown) for delivery of the GTD into the cytosol. **B** Domain architecture of CDTa and CDTb. CDTa consists of an N-terminal pADPRT, pseudo-ADP-ribosyltransferase (pADPRT) followed by an active ADP-ribosyltransferase (ADPRT) that modifies G-actin monomers upon entry into the host cell cytosol via CDTb, which consists of the domains D1 (activation by host proteases), D2/D3 (insertion and pore formation in endosomal membranes upon acidification of the endosomes via vacuolar H^+^-ATPases (shown in brown) for delivery of CDTa into the cytosol), D3 (oligomerization), and D4 (receptor binding; including a glycan-binding domain (GBD)). **C** Cellular uptake, mode of action, and inhibitors of TcdB (exemplarily; left part) and CDT (right part). The four steps in Roman numerals indicate I, receptor binding (for CDT receptor binding is followed by host protease-mediated cleavage and activation at the D1 domain, oligomerization at the plasma membrane by the D3 domain and recruitment of CDTa to the receptor: CDTb oligomer complex); I*, direct pore formation of the CDTb oligomer into the plasma membrane; II, receptor-mediated endocytosis; III, endosome-to-cytosol translocation of the enzyme domains (GTD and CDTa, respectively) and refolding with the help of host chaperons; IV, modification of target substrates (GTD, glucosylation of Rho and/or Ras GTPases, e.g., Rac1; CDTa, ADP-ribosylation of G-actin). Inhibitory drugs and the affected steps during cell entry of the toxins are indicated by red boxes and dashed arrows. 3D structures of TcdB (PDB ID: 6OQ5; Chen et al. 2019) and of Rac1 (PDB ID: 3TH5; Krauthammer et al. 2012) as well as 3D structures of the CDTb oligomer in the prepore conformation in complex or without CDTa (PDB ID: 6V1S; Sheedlo et al. 2020), of the CDTb pore with long stem in complex with CDTa (PDB ID: 7VNN, Kawamoto et al. 2022), of CDTa (PDB ID: 2WN4; Sundriyal et al. 2009), and of G-actin (PDB ID: 2HF3; Rould et al. 2006) were generated with Mol* (Sehnal et al. 2021). Membrane-inserting structures of CDTb at the plasma membrane and TcdB in endosomal membrane are fictitious and for representation only. Abbreviations: InsP6, inositol hexakisphosphate; Glc, glucose; LSR, lipolysis-stimulated lipoprotein receptor

TcdA and TcdB (shortly TcdA/B) are independently acting, single-chain toxins with a rather large size of 308 (TcdA) and 270 kD (TcdB), respectively. They consist of several functional domains whose orchestrated interplay is required for the delivery of the toxic cargo, an N-terminally located glucosyltransferase domain, into target cells (Aktories et al. 2017). The domain architecture of TcdA/B and their functions at consecutive steps during cell entry of TcdA/B are depicted in detail in Fig. 3A.

The target molecules of TcdA/B are small GTPases of the Rho and/or Ras family (Just et al. 1995a, b; Just & Gerhard 2004; Genth et al. 2018; Zeiser et al. 2013), which act in cells as molecular switches and as master regulators of the actin cytoskeleton and of numerous other cellular processes, including cell migration, phagocytosis, intracellular trafficking, cell progression, and apoptosis (Nobes & Hall 1994; Burridge and Wennerberg 2004 l; Jaffe & Hall 2005; Aktories 2011; Lemichez & Aktories 2013). TcdA and TcdB vary in their substrate profile but they both inactivate their substrates by covalent attachment of a glucose moiety at a conserved threonine residue, which is crucial for the interaction with effectors. The glucose is provided by UDP-glucose, which acts as a co-substrate for the toxins’ glucosyltransferase domain. In this respect, TcdA/B are members of the family of clostridial glucosylating toxins (CGTs) (Jank & Aktories 2008), also denoted as the family of large clostridial cytotoxins (LCCs) (von Eichel-Streiber et al. 1996).

TcdA/B-mediated glucosylation of Rho proteins causes a number of changes in cellular function, but cell rounding is the most obvious cytopathological effect in cultured mammalian cells. Due to a redistribution of the actin cytoskeleton, the cells lose their normal shape and form irregular extensions, a process which is called arborization. The role of the toxins during CDI pathogenesis is not entirely understood. However, pathophysiological effects of the toxins, such as disruption of the barrier function of enterocytes, impairing colonic epithelial renewal, increasing colonic vascular permeability, induction of apoptosis, and pro-inflammatory activities, are altogether contributing and promoting disease pathogenesis. The numerous cytopathological and pathophysiological effects of TcdA/B were summarized recently in detail (Papatheodorou et al. 2018).

#### Structure, uptake, mode of action, and pathophysiological role of CDT

CDT is binary toxin formed by two separate components, the binding and translocation component CDTb and the enzyme component CDTa. The toxin is highly similar to other clostridial binary ADP-ribosylating toxins, such as the C2 toxin from *Clostridium botulinum* and the iota-toxin from *Clostridium perfringens*, and more distantly related to the anthrax toxin of *Bacillus anthracis* (Stiles et al. 2014; Aktories et al. 2018). CDTb binds to its receptor, the lipolysis-stimulated lipoprotein receptor (LSR) (Papatheodorou et al. 2011), and upon entry into endosomes, and facilitates by forming oligomeric pores the translocation of CDTa into the cytosol. In the cytosol, CDTa utilizes NAD for covalent attachment of an ADP-ribose moiety to monomeric G-actin. ADP-ribosylated G-actin monomers block the polymerization of F-actin filaments, which eventually leads to the collapse of the actin cytoskeleton. Figure 3B illustrates the modular composition of CDT and its various steps during cell entry. Recent findings indicate that CDTb alone is also capable of damaging cells by pore formation in the plasma membrane (Landenberger et al. 2021).

The pathophysiological role of CDT still remains enigmatic. However, a recent retrospective study has shown that CDT-positive patients were associated with increased disease severity and worse clinical outcomes (Young et al. 2022). One explanation might be that CDT increases the adherence of the *C. difficile* bacteria at the surface of intestinal epithelial cells. CDT-induced disruption of the actin cytoskeleton leads to the formation of long microtubule-based protrusions on the surface of intestinal host cells. These protrusions enwrap the bacteria, resulting in increased pathogen adherence (Schwan et al. 2009).

### Novel pharmacological approaches against *C. difficile* toxins

The growing mechanistic knowledge about the biology of *C. difficile* toxins has led to novel anti-toxin approaches, which might be useful in the future as supportive treatment options against *C. difficile*-associated diseases and/or post-traumatic complications. For instance, body-own antimicrobial peptides, such as certain defensins, were shown to inhibit TcdA/B and CDT either by direct interaction and formation of biologically inactive aggregates (Fischer et al. 2020; Korbmacher et al. 2020; Barthold et al. 2022) or by inhibiting enzyme activities, as suggested for human α-defensin and TcdB (Giesemann et al. 2008).

Along with body-own or artificial peptide libraries, “drug repurposing” (also known as “drug repositioning”) is another promising approach for the discovery of novel pharmacological approaches against *C. difficile* toxins on the basis of already licensed and safe-to-use drugs. It is a cost-effective and time-efficient way to develop new anti-toxin treatments with a high probability of success.

Since now, several licensed drugs have been found that exhibits activity against *C. difficile* toxins. The mucolytic agent ambroxol, for instance, has been shown to specifically inhibit the glucosyltransferase activity of TcdA/B (Heber et al. 2022). The antiemetic and prokinetic drug domperidone inhibits the refolding of the glucosyltransferase domain of TcdA/B and of the enzyme component CDTa after the translocation from endosomes as linear, unfolded proteins into the cytosol (Braune-Yan et al. 2023). In this context, domperidone acts as an inhibitor of Hsp70 (Concilli et al. 2020), a chaperone crucially involved in the refolding step during cell entry of TcdA/B and CDT (Ernst 2022; Braune-Yan et al. 2023).

Pore formation of TcdA/B is another critical step during cell entry and requires the presence of cholesterol in endosomal membranes. Therefore, the hypocholesterolemic drug simvastatin, which acts as an inhibitor of the HMG-CoA reductase, was found to be capable of inhibiting cell entry of TcdA/B by decreasing the cholesterol content in membranes of cultured cells (Papatheodorou et al. 2019). Recently, it was shown that the antiarrhythmic drug amiodarone prevents intoxication of cells by TcdA/B (Schumacher et al. 2023). Amiodarone’s main mode of inhibition likely involves interference with pore formation and translocation of both toxins.

For CDT, not only domperidone, but also the antibacterial drug bacitracin and the immunosuppressant cyclosporine A have been proven to interfere with the endosome-to-cytosol translocation of CDTa and to thus inhibit the intoxication of cells with CDT (Schnell et al. 2019). Interestingly, later, it was found that bacitracin was also effective against TcdB, most likely due to inhibition of the translocation of the glucosyltransferase domain across the endosomal membrane (Zhu et al. 2019). Another approach for inhibiting CDT is the direct blockage of its pore formed by CDTb with chemical compounds. The antimalarial drug chloroquine is among various substances that are capable of inhibiting the CDTb pore, thereby preventing not only the endosome-to-cytosol translocation of CDTa, but also the cytotoxic effects, which are associated with CDTb-dependent pore formation at the plasma membrane (Ernst et al. 2021). Licensed drugs capable of inhibiting TcdA/B and/or CDT and their proposed mode of inhibition are summarized in Fig. 3C.

## Conclusions

Trauma-toxicology, traumatology, and toxicology are related medical fields, but they have distinct focuses and approaches (see Table 4). Trauma-toxicology is a specialized area of toxicology that focuses on the effects of toxic substances on injured patients, while traumatology focuses on the prevention, diagnosis, and treatment of injuries (see Table 3).

**Table 3:** Comparison between traumatology, toxicology, and trauma-toxicology

	Traumatology	Toxicology	Trauma-toxicology
Focus	Special, well-established medical field: prevention, diagnosis, and treatment of injuries	Well-established research field (experimental toxicology), medical field (clinical toxicology), and regulation of toxic compounds (regulatory toxicology)	Quite novel research and medical field that combines certain aspects of traumatology and toxicology: effects of toxic substances on injured patients
Impact	Defined spatio-temporal trauma vector	Hidden or evident exposure to toxic substances	Trauma vector plus toxic exposure or toxic complication
Diagnostics	Imaging – easy, fast (within the golden hour)	Difficult, time-consuming	Difficult, often highly specific (e.g., B- and C-warfare), time-consuming, often unclear, sophisticated, and robust imaging required
Triggers	Mainly DAMPs, PAMPs, bacterial toxins as complication	PAMPs, bacterial toxins, warfare toxins	DAMPs, PAMPs, bacterial toxins, toxic agents (e.g., B- and C-warfare)
Host pathophysiology	Barrier damage, immune disfunction, organ disfunction	Barrier damage, immune dysfunction, organ disfunction	Barrier damage, immune dysfunction, organ disfunction
Treatment	Surgical damage control, causative	Removal/decontamination, drugs/antidotes/symptomatic	Complex: multidisciplinary surgical plus removal/decontamination plus drug/antidotes/symptomatic
Prognosis	Good – depending on the injury severity	Medium—depending on the nature and dose of the toxic agent(s)	Mainly poor—depending on injury severity plus diagnostic and treatment resources, and the nature and dose of toxic agent(s)
Research efforts	Frequent, broad	Some expertise (mainly research activities)	Rare (mainly DOD-driven)

In conclusion, for the first time, we coin and define the term “trauma-toxicology” referring the interplay and “vicious circle” of severe tissue trauma, barrier breakdown, pathogen and toxin exposure, and subsequent tissue damage. Furthermore, trauma can be associated with toxins (e.g., biological warfare) and toxic agents (e.g., chemical warfare) leading also to macro- and micro-barrier break down and the “vicious circle” and frequently often fatal outcome. In this context, toxins can be the reason for the trauma or essentially contribute to post-traumatic complications after traumatic injuries. Although the underlying cellular and molecular mechanisms of both toxins and toxic agents are well described for most substances, treatment options need further research and translation to the real world in order to sufficiently improve trauma-toxicological conditions. Some keyterms in the field of “trauma-toxicology” are defined in Table 4.

**Table 4:** Definition of key terms in trauma-toxicology

Term	Definition, meaning
Pathobiome	The microbiome, which underwent a shift towards the growth of more pathogenic bacteria in the gut. This might be induced by prolonged treatment with antibacterial drugs or by traumatic injuries
Time until first treatment	The time elapsed till the first professional diagnostic measures and treatment is induced
Macrocosm	The patient
Microcosm	The microbiome of a patient
B-warfare	Biological warfare, e.g., viruses, bacteria, toxins from bacteria (anthrax toxins, botulinum neurotoxin) or plants (ricin)
C-warfare	Chemical warfare (see Table 1)
Post-traumatic complications	Clinical complications in patients after traumatic injury, such as infections with toxin-producing bacteria in hospital (e.g., *C. difficile*, *Staphylococcus aureus*, *Streptococcus pyogenes*) that may worsen the outcome of these trauma patients
Trigger-to-threat	Manifestation of characteristic clinical symptoms after exposure to a “trigger” (e.g., a toxic substance)
Intoxicated trauma patient	A patient that has an intoxication, e.g., by B- or C-warfare in addition to the traumatic injuries

## Data Availability

Not applicable.

## Abbreviations

AC: Hydrogen cyanide
AChE: Acetylcholinesterase
ADP: Ribosyltransferase
B-warfare: Biological warfare
C.: *Clostridioides*
CBRN: Chemical, biological or radiological/nuclear warfare agents
CDAD: *C. difficile*-Associated disease
CDI: *C. difficile*-Induced infection
CDT: *C. difficile* Transferase
ADPRT: CG, phosgene
CGTs: Clostridial glucosylating toxins
CK: Cyanogen chloride (CK)
Cl: Chlorine
CNS: Central nerve system
C-warfare: Chemical warfare
CWAs: Chemical warfare agents
DAMPs: Damage-associated molecular patterns
GA: Tabun
GB: Sarin
GBB: Gut-blood-barrier
GD: Soman
GTPases: Guanine nucleotide-binding proteins (G-proteins)
ICU: Intensive care unit
ISS: Injury severity score
LC: Liquid chromatography
LCCs: Large clostridial cytotoxins
MODS: Multiple organ dysfunction syndrome
MS: Mass spectrometry
OP: Organophosphate
OPNA: Organophosphorus nerve agent
PAMPs: Pathogen-associated molecular patterns
PS: Chloropicrin
SEA: Enterotoxin A from *Staphylococcus aureus*
SEB: Enterotoxin B from *Staphylococcus aureus*
TBI: Mild traumatic brain injury
TcdA: Toxin A from *C. difficile*
TcdB: Toxin B from *C. difficile*
TSST-1: Toxic shock syndrome toxin-1
WWI: World War I

## References

[CR1] Abrams ST, Wang L, Yong J, Yu Q, Du M, Alhamdi Y, Cheng Z, Dart C, Lane S, Yu W, Toh CH, Wang G (2022). The importance of pore-forming toxins in multiple organ injury and dysfunction. Biomedicines.

[CR2] Aktories K (2011). Bacterial protein toxins that modify host regulatory GTPases. Nat Rev Microbiol.

[CR3] Aktories K, Schwan C, Jank T (2017). Clostridium difficile toxin biology. Annu Rev Microbiol.

[CR4] Aktories K, Papatheodorou P, Schwan C (2018). Binary Clostridium difficile toxin (CDT) - a virulence factor disturbing the cytoskeleton. Anaerobe.

[CR5] Ali RA, Khan MA, Anjum AA, Khubaib Sattar MM, Sarwar A, Ali T, Tariq M, Iqbal A (2022). Molecular markers for the detection of pathogenic and food poisoning potential of methicillin resistant Staphylococcus aureus isolated from wounds of hospitalized patients. Pak J Pharm Sci.

[CR6] Amend N, Niessen KV, Seeger T, Wille T, Worek F, Thiermann H (2020). Diagnostics and treatment of nerve agent poisoning-current status and future developments. Ann N Y Acad Sci.

[CR7] Appiah SA, Foxx CL, Langgartner D, Palmer A, Zambrano CA, Braumuller S, Schaefer EJ, Wachter U, Elam BL, Radermacher P, Stamper CE, Heinze JD, Salazar SN, Luthens AK, Arnold AL, Reber SO, Huber-Lang M, Lowry CA, Halbgebauer R (2021). Evaluation of the gut microbiome in association with biological signatures of inflammation in murine polytrauma and shock. Sci Rep.

[CR8] Armacki M, Trugenberger AK, Ellwanger AK, Eiseler T, Schwerdt C, Bettac L, Langgartner D, Azoitei N, Halbgebauer R, Gross R, Barth T, Lechel A, Walter BM, Kraus JM, Wiegreffe C, Grimm J, Scheffold A, Schneider MR, Peuker K, Zeissig S, Britsch S, Rose-John S, Vettorazzi S, Wolf E, Tannapfel A, Steinestel K, Reber SO, Walther P, Kestler HA, Radermacher P, Barth TF, Huber-Lang M, Kleger A, Seufferlein T (2018). Thirty-eight-negative kinase 1 mediates trauma-induced intestinal injury and multi-organ failure. J Clin Invest.

[CR9] Bansal V, Costantini T, Ryu SY, Peterson C, Loomis W, Putnam J, Elicieri B, Baird A, Coimbra R (2010). Stimulating the central nervous system to prevent intestinal dysfunction after traumatic brain injury. J Trauma.

[CR10] Barthold L, Heber S, Schmidt CQ, Gradl M, Weidinger G, Barth H, Fischer S (2022). Human α-defensin-6 neutralizes clostridioides difficile toxins TcdA and TcdB by direct binding. Int J Mol Sci.

[CR11] Black R, Worek F, Jenner J, Thiermann H (2016). Development, Historical Use and Properties of Chemical Warfare Agents. Chemical warfare toxicology.

[CR12] Bowman WC (2006). Neuromuscular block. Br J Pharmacol.

[CR13] Braune-Yan M, Jia J, Wahba M, Schmid J, Papatheodorou P, Barth H, Ernst K (2023). Domperidone protects cells from intoxication with Clostridioides difficile toxins by inhibiting Hsp70-assisted membrane translocation. Toxins.

[CR14] Brunka Z, Ryl J, Brushtulli P, Gromala D, Walczak G, Zieba S, Piesniak D, Sein Anand J, Wiergowski M (2022). Selected political criminal poisonings in the years 1978–2020: detection and treatment. Toxics.

[CR15] Burk AM, Martin M, Flierl MA, Rittirsch D, Helm M, Lampl L, Bruckner U, Stahl GL, Blom AM, Perl M, Gebhard F, Huber-Lang M (2012). Early complementopathy after multiple injuries in humans. Shock.

[CR16] Burridge K, Wennerberg K (2004). Rho and Rac take center stage. Cell.

[CR17] Chen P, ho Lam K, Liu Z, Mindlin FA, Chen B, Gutierrez CB, Huang L, Zhang Y, Hamza T, Feng H, Matsui T, Bowen ME, Perry K, Jin R (2019). Structure of the full-length Clostridium difficile toxin B. Nat Struct Mol Biol.

[CR18] Concilli M, Petruzzelli R, Parisi S, Catalano F, Sirci F, Napolitano F, Renda M, Galietta LJV, Bernardo DD, Polishchuk RS (2020). Pharmacoproteomics pinpoints HSP70 interaction for correction of the most frequent Wilson disease-causing mutant of ATP7B. Proc Natl Acad Sci USA.

[CR19] Deitch EA, Feketeova E, Adams JM, Forsythe RM, Xu DZ, Itagaki K, Redl H (2006). Lymph from a primate baboon trauma hemorrhagic shock model activates human neutrophils. Shock.

[CR20] Depestel DD, Aronoff DM (2013). Epidemiology of Clostridium difficile infection. J Pharm Pract.

[CR21] Ernst K, Sailer J, Braune M, Barth H (2021). Intoxication of mammalian cells with binary clostridial enterotoxins is inhibited by the combination of pharmacological chaperone inhibitors. Naunyn-Schmiedeberg’s Arch Pharmacol.

[CR22] Ernst, K (2022) Requirement of peptidyl-prolyl Cis/Trans isomerases and chaperones for cellular uptake of bacterial AB-type toxins. Front Cellular Infect Microbiol, 12. 10.3389/FCIMB.2022.938015 10.3389/fcimb.2022.938015 PMC9387773 35992160

[CR23] Fang JF, Shih LY, Yuan KC, Fang KY, Hwang TL, Hsieh SY (2010). Proteomic analysis of post-hemorrhagic shock mesenteric lymph. Shock.

[CR24] Faries PL, Simon RJ, Martella AT, Lee MJ, Machiedo GW (1998). Intestinal permeability correlates with severity of injury in trauma patients. J Trauma.

[CR25] Feighery L, Smyth A, Keely S, Baird AW, O’Connor WT, Callanan JJ, Brayden DJ (2008). Increased intestinal permeability in rats subjected to traumatic frontal lobe percussion brain injury. J Trauma.

[CR26] Fischer S, Ückert AK, Landenberger M, Papatheodorou P, Hoffmann-Richter C, Mittler AK, Ziener U, Hägele M, Schwan C, Müller M, Kleger A, Benz R, Popoff MR, Aktories K, Barth H (2020). Human peptide α-defensin-1 interferes with Clostridioides difficile toxins TcdA, TcdB, and CDT. FASEB J.

[CR27] Ganesan K, Raza SK, Vijayaraghavan R (2010). Chemical warfare agents. J Pharm Bioallied Sci.

[CR28] Genth H, Junemann J, Lämmerhirt CM, Lücke A-C, Schelle I, Just I, Gerhard R, Pich A (2018). Difference in mono-O-glucosylation of Ras subtype GTPases between toxin A and toxin B from Clostridioides difficile strain 10463 and lethal toxin from Clostridium sordellii strain 6018. Front Microbiol.

[CR29] Giesemann T, Guttenberg G, Aktories K (2008). Human alpha-defensins inhibit Clostridium difficile toxin B. Gastroenterol.

[CR30] Gonzalez CA, Van Rysselberghe NL, Maschhoff C, Gardner MJ (2022). Clostridium difficile colitis portends poor outcomes in lower extremity orthopaedic trauma surgery. Injury.

[CR31] Haffner-Luntzer M, Foertsch S, Fischer V, Prystaz K, Tschaffon M, Modinger Y, Bahney CS, Marcucio RS, Miclau T, Ignatius A, Reber SO (2019). Chronic psychosocial stress compromises the immune response and endochondral ossification during bone fracture healing via beta-AR signaling. Proc Natl Acad Sci U S A.

[CR32] Halbgebauer R, Braun CK, Denk S, Mayer B, Cinelli P, Radermacher P, Wanner GA, Simmen HP, Gebhard F, Rittirsch D, Huber-Lang M (2018). Hemorrhagic shock drives glycocalyx, barrier and organ dysfunction early after polytrauma. J Crit Care.

[CR33] Heber S, Barthold L, Baier J, Papatheodorou P, Fois G, Frick M, Barth H, Fischer S (2022). Inhibition of Clostridioides difficile toxins TcdA and TcdB by Ambroxol. Front Pharmacol.

[CR34] Huber-Lang M, Lambris JD, Ward PA (2018). Innate immune responses to trauma. Nat Immunol.

[CR35] Jachowicz E, Pac A, Rozanska A, Gryglewska B, Wojkowska-Mach J (2022). Post-discharge Clostridioides difficile infection after arthroplasties in Poland, infection prevention and control as the key element of prevention of C. difficile infections. Int J Environ Res Public Health.

[CR36] Jaffe AB, Hall A (2005). Rho GTPases: biochemistry and biology. Annu Rev Cell Dev Biol.

[CR37] Jank T, Aktories K (2008). Structure and mode of action of clostridial glucosylating toxins: the ABCD model. Trends Microbiol.

[CR38] John H, Siegert M, Eyer F, Worek F, Thiermann H, Kranawetvogl A (2018). Novel cysteine- and albumin-adduct biomarkers to prove human poisoning with the pesticide oxydemeton-S-methyl. Toxicol Lett.

[CR39] John H, Thiermann H (2021). Poisoning by organophosphorus nerve agents and pesticides: an overview of the principle strategies and current progress of mass spectrometry-based procedures for verification. J Mass Spectrom Adv Clin Lab.

[CR40] Just I, Gerhard R (2004). Large clostridial cytotoxins. Rev Physiol Biochem Pharmacol.

[CR41] Just I, Selzer J, Wilm M, Eichel-Streiber CV, Mann M, Aktories K (1995). Glucosylation of Rho proteins by Clostridium difficile toxin B. Nature.

[CR42] Just I, Wilm M, Selzer J, Rex G, Von Eichel-Streiber C, Mann M, Aktories K (1995). The enterotoxin from Clostridium difficile (ToxA) monoglucosylates the Rho proteins. J Biol Chem.

[CR43] Kaempfer R, Arad G, Levy R, Hillman D (2002). Defense against biologic warfare with superantigen toxins. Isr Med Assoc J.

[CR44] Karamanos E, Gupta AH, Stanton CN, Mohamed A, Patton JH, Schmoekel N (2018). Clostridium difficile-associated infection in trauma patients: development of the Clostridium difficile influencing factors (CDIF) score. Perm J.

[CR45] Kawamoto, A, Yamada, T, Yoshida, T, Sato, Y, Kato, T, Tsuge, H (2022) Cryo-EM structures of the translocational binary toxin complex CDTa-bound CDTb-pore from Clostridioides difficile. Nature Communications, 13(1). 10.1038/s41467-022-33888-4 10.1038/s41467-022-33888-4 PMC9576733 36253419

[CR46] Khoshnevis MA, Panahi Y, Ghanei M, Borna H, Sahebkar A, Aslani J (2015) A triage model for chemical warfare casualties. Trauma Mon 20(3):e16211. 10.5812/traumamon.16211 10.5812/traumamon.16211 PMC4630593 26543836

[CR47] Korbmacher, M, Fischer, S, Landenberger, M, Papatheodorou, P, Aktories, K, Barth, H (2020) Human α-defensin-5 efficiently neutralizes Clostridioides difficile toxins TcdA, TcdB, and CDT. Frontiers in Pharmacology, 11. 10.3389/FPHAR.2020.01204 10.3389/fphar.2020.01204 PMC7435013 32903430

[CR48] Kranawetvogl T, Kranawetvogl A, Scheidegger L, Wille T, Steinritz D, Worek F, Thiermann H, John H (2023). Evidence of nerve agent VX exposure in rat plasma by detection of albumin-adducts in vitro and in vivo. Arch Toxicol.

[CR49] Krauthammer M, Kong Y, Ha BH, Evans P, Bacchiocchi A, McCusker JP, Cheng E, Davis MJ, Goh G, Choi M, Ariyan S, Narayan D, Dutton-Regester K, Capatana A, Holman EC, Bosenberg M, Sznol M, Kluger HM, Brash DE, Halaban R (2012). Exome sequencing identifies recurrent somatic RAC1 mutations in melanoma. Nat Genet.

[CR50] Kreyden OP, Geiges ML, Boni R, Burg G (2000). Botulinum toxin: from poison to drug. A Historical Review Hautarzt.

[CR51] Landenberger M, Nieland J, Roeder M, Nørgaard K, Papatheodorou P, Ernst K, Barth H (2021). The cytotoxic effect of Clostridioides difficile pore-forming toxin CDTb. Biochimica et Biophysica Acta - Biomembranes.

[CR52] Langgartner D, Vaihinger CA, Haffner-Luntzer M, Kunze JF, Weiss AJ, Foertsch S, Bergdolt S, Ignatius A, Reber SO (2018). The role of the intestinal microbiome in chronic psychosocial stress-induced pathologies in male mice. Front Behav Neurosci.

[CR53] Lemichez E, Aktories K (2013). Hijacking of Rho GTPases during bacterial infection. Exp Cell Res.

[CR54] Levy G, Fishman JE, Xu D, Chandler BT, Feketova E, Dong W, Qin Y, Alli V, Ulloa L, Deitch EA (2013). Parasympathetic stimulation via the vagus nerve prevents systemic organ dysfunction by abrogating gut injury and lymph toxicity in trauma and hemorrhagic shock. Shock.

[CR55] Lumpkins K, Bochicchio GV, Joshi M, Gens R, Bochicchio K, Conway A, Schaub S, Scalea T (2008). Clostridium difficile infection in critically injured trauma patients. Surg Infect (larchmt).

[CR56] Ma Y, Liu T, Fu J, Fu S, Hu C, Sun B, Fan X, Zhu J (2019). Lactobacillus acidophilus Exerts Neuroprotective Effects in Mice with Traumatic Brain Injury. J Nutr.

[CR57] Magnotti LJ, Upperman JS, Xu DZ, Lu Q, Deitch EA (1998). Gut-derived mesenteric lymph but not portal blood increases endothelial cell permeability and promotes lung injury after hemorrhagic shock. Ann Surg.

[CR58] Maitz A, Haussner F, Braumuller S, Hoffmann A, Lupu L, Wachter U, Radermacher P, Braun CK, Wilke HJ, Vogt M, Ignatius A, Halbgebauer R, Bettac L, Barth TFE, Huber-Lang M, Palmer A (2021). Temporal-spatial organ response after blast-induced experimental blunt abdominal trauma. FASEB J.

[CR59] Meier K, Nordestgaard AT, Eid AI, Kongkaewpaisan N, Lee JM, Kongwibulwut M, Han KR, Kokoroskos N, Mendoza AE, Saillant N, King DR, Velmahos GC, Kaafarani HMA (2019). Obesity as protective against, rather than a risk factor for, postoperative Clostridium difficile infection: a nationwide retrospective analysis of 1,426,807 surgical patients. J Trauma Acute Care Surg.

[CR60] Mino MJ, Ortiz RT, Randad P, Moffatt LT, Jordan MH, Shupp JW (2013). Localization of superantigen virulence factors in kidney tissue of animals with Staphylococcus aureus-infected burn wounds. J Burn Care Res.

[CR61] Munley JA, Kelly LS, Pons EE, Kannan KB, Coldwell PS, Whitley EM, Gillies GS, Efron PA, Nagpal R, Mohr AM (2023). Multicompartmental traumatic injury and the microbiome: shift to a pathobiome. J Trauma Acute Care Surg.

[CR62] Nicholson SE, Merrill D, Zhu C, Burmeister DM, Zou Y, Lai Z, Darlington DN, Lewis AM, Newton L, Scroggins S, Eastridge BJ, Schwacha MG (2018). Polytrauma independent of therapeutic intervention alters the gastrointestinal microbiome. Am J Surg.

[CR63] Nobes C, Hall A (1994). Regulation and function of the Rho subfamily of small GTPases. Curr Opin Genet Dev.

[CR64] Olson A, Diebel LN, Liberati DM (2014) Exogenous phosphatidylcholine supplementation improves intestinal barrier defense against Clostridium difficile toxin. J Trauma Acute Care Surg 77:570–575. 10.1097/TA.0000000000000378 10.1097/TA.0000000000000378 25250596

[CR65] Papatheodorou P, Carette JE, Bell GW, Schwan C, Guttenberg G, Brummelkamp TR, Aktories K (2011). Lipolysis-stimulated lipoprotein receptor (LSR) is the host receptor for the binary toxin Clostridium difficile transferase (CDT). Proc Natl Acad Sci USA.

[CR66] Papatheodorou P, Barth H, Minton N, Aktories K (2018). Cellular uptake and mode-of-action of Clostridium difficile toxins. Adv Exp Med Biol.

[CR67] Papatheodorou P, Song S, López-Ureña D, Witte A, Marques F, Ost GS, Schorch B, Chaves-Olarte E, Aktories K (2019). Cytotoxicity of Clostridium difficile toxins A and B requires an active and functional SREBP-2 pathway. FASEB J.

[CR68] Pinchuk IV, Beswick EJ, Reyes VE (2019). Staphylococcal enterotoxins. Toxins (Basel)..

[CR69] Prindeze NJ, Amundsen BM, Pavlovich AR, Paul DW, Carney BC, Moffatt LT, Shupp JW (2014). Staphylococcal superantigens and toxins are detectable in the serum of adult burn patients. Diagn Microbiol Infect Dis.

[CR70] Rossaint R, Afshari A, Bouillon B, Cerny V, Cimpoesu D, Curry N, Duranteau J, Filipescu D, Grottke O, Gronlykke L, Harrois A, Hunt BJ, Kaserer A, Komadina R, Madsen MH, Maegele M, Mora L, Riddez L, Romero CS, Samama CM, Vincent JL, Wiberg S, Spahn DR (2023) The European guideline on management of major bleeding and coagulopathy following trauma: sixth edition. Crit Care 27: 80. 10.1186/s13054-023-04327-7 10.1186/s13054-023-04327-7 PMC9977110 36859355

[CR71] Rould MA, Wan Q, Joel PB, Lowey S, Trybus KM (2006). Crystal structures of expressed non-polymerizable monomeric actin in the ADP and ATP states. J Biol Chem.

[CR72] Sartelli M, Di Bella S, McFarland LV, Khanna S, Furuya-Kanamori L, Abuzeid N, Abu-Zidan FM, Ansaloni L, Augustin G, Bala M, Ben-Ishay O, Biffl WL, Brecher SM, Camacho-Ortiz A, Cainzos MA, Chan S, Cherry-Bukowiec JR, Clanton J, Coccolini F, Cocuz ME, Coimbra R, Cortese F, Cui Y, Czepiel J, Demetrashvili Z, Di Carlo I, Di Saverio S, Dumitru IM, Eckmann C, Eiland EH, Forrester JD, Fraga GP, Frossard JL, Fry DE, Galeiras R, Ghnnam W, Gomes CA, Griffiths EA, Guirao X, Ahmed MH, Herzog T, Kim JI, Iqbal T, Isik A, Itani KMF, Labricciosa FM, Lee YY, Juang P, Karamarkovic A, Kim PK, Kluger Y, Leppaniemi A, Lohsiriwat V, Machain GM, Marwah S, Mazuski JE, Metan G, Moore EE, Moore FA, Ordonez CA, Pagani L, Petrosillo N, Portela F, Rasa K, Rems M, Sakakushev BE, Segovia-Lohse H, Sganga G, Shelat VG, Spigaglia P, Tattevin P, Trana C, Urbanek L, Ulrych J, Viale P, Baiocchi GL, Catena F (2019). 2019 update of the WSES guidelines for management of Clostridioides (Clostridium) difficile infection in surgical patients. World J Emerg Surg.

[CR73] Sartelli M, Ansaloni L, Biffl WA, Coccolini F, De Simone B, Leppaniemi A, Kluger Y, Tolonen M, Moore EE, Catena F (2021). World Society of Emergency Surgery-American Association for the Surgery of Trauma Guidelines for management of Clostridioides (Clostridium) difficile infection in surgical patients: an executive summary. J Trauma Acute Care Surg.

[CR74] Schnell L, Felix I, Müller B, Sadi M, Von Bank F, Papatheodorou P, Popoff MR, Aktories K, Waltenberger E, Benz R, Weichbrodt C, Fauler M, Frick M, Barth H (2019). Revisiting an old antibiotic: bacitracin neutralizes binary bacterial toxins and protects cells from intoxication. FASEB J Off Publ Fed Am Soc Exp Biol.

[CR75] Schuijt TJ, van der Poll T, de Vos WM, Wiersinga WJ (2013). The intestinal microbiota and host immune interactions in the critically ill. Trends Microbiol.

[CR76] Schumacher J, Nienhaus A, Heber S, Matylitsky J, Chaves-Olarte E, Rodríguez C, Barth H, Papatheodorou P (2023). Exploring the inhibitory potential of the antiarrhythmic drug amiodarone against Clostridioides difficile toxins TcdA and TcdB. Gut Microbes.

[CR77] Schwan C, Stecher B, Tzivelekidis T, Van Ham M, Rohde M, Hardt WD, Wehland J, Aktories K (2009). Clostridium difficile toxin CDT induces formation of microtubule-based protrusions and increases adherence of bacteria. PLoS Pathogens.

[CR78] Sehnal D, Bittrich S, Deshpande M, Svobodová R, Berka K, Bazgier V, Velankar S, Burley SK, Koča J, Rose AS (2021). Mol* Viewer: modern web app for 3D visualization and analysis of large biomolecular structures. Nucleic Acids Res.

[CR79] Sgro M, Iacono G, Yamakawa GR, Kodila ZN, Marsland BJ, Mychasiuk R (2022). Age matters: microbiome depletion prior to repeat mild traumatic brain injury differentially alters microbial composition and function in adolescent and adult rats. PLoS ONE.

[CR80] Sheedlo MJ, Anderson DM, Thomas AK, Borden Lacy D (2020). Structural elucidation of the Clostridioides difficile transferase toxin reveals a single-site binding mode for the enzyme. Proc Natl Acad Sci USA.

[CR81] Shoaei P, Shojaei H, Siadat SD, Moshiri A, Vakili B, Yadegari S, Ataei B, Khorvash F (2022). Gut microbiota in burned patients with Clostridioides difficile infection. Burns.

[CR82] Spindler-Vesel A, Wraber B, Vovk I, Kompan L (2006). Intestinal permeability and cytokine inflammatory response in multiply injured patients. J Interferon Cytokine Res.

[CR83] Stiles BG, Pradhan K, Fleming JM, Samy RP, Barth H, Popoff MR (2014). Clostridium and bacillus binary enterotoxins: bad for the bowels, and eukaryotic being. Toxins.

[CR84] Sundriyal A, Roberts AK, Shone CC, Acharya KR (2009). Structural basis for substrate recognition in the enzymatic component of ADP-ribosyltransferase toxin CDTa from Clostridium difficile. J Biol Chem.

[CR85] Tin D, Sabeti P, Ciottone GR (2022). Bioterrorism: an analysis of biological agents used in terrorist events. Am J Emerg Med.

[CR86] Valido E, Bertolo A, Frankl GP, Itodo OA, Pinheiro T, Pannek J, Kopp-Heim D, Glisic M, Stoyanov J (2022). Systematic review of the changes in the microbiome following spinal cord injury: animal and human evidence. Spinal Cord.

[CR87] Vanzant EL, Ozrazgat-Baslanti T, Liu H, Malik S, Davis R, Lanz J, Miggins MV, Gentile LF, Cuenca A, Cuenca AG, Lottenberg L, Moore FA, Ang DN, Bihorac A, Efron PA (2015). Clostridium difficile infections after blunt trauma: a different patient population?. Surg Infect (larchmt).

[CR88] Von Eichel-Streiber C, Boquet P, Sauerborn M, Thelestam M (1996). Large clostridial cytotoxins–a family of glycosyltransferases modifying small GTP-binding proteins. Trends Microbiol.

[CR89] Walsh DM, McCullough SD, Yourstone S, Jones SW, Cairns BA, Jones CD, Jaspers I, Diaz-Sanchez D (2017). Alterations in airway microbiota in patients with PaO2/FiO2 ratio </= 300 after burn and inhalation injury. PLoS ONE.

[CR90] Wheatley EG, Curtis BJ, Hulsebus HJ, Boe DM, Najarro K, Ir D, Robertson CE, Choudhry MA, Frank DN, Kovacs EJ (2020). Advanced age impairs intestinal antimicrobial peptide response and worsens fecal microbiome dysbiosis following burn injury in mice. Shock.

[CR91] Wille T, Thiermann H, Worek F (2011). In vitro kinetic interactions of DEET, pyridostigmine and organophosphorus pesticides with human cholinesterases. Chem Biol Interact.

[CR92] Wille T, Steinritz D, Worek F, Thiermann H (2019). Chemical warfare agent poisoning]. Bundesgesundheitsblatt Gesundheitsforschung Gesundheitsschutz.

[CR93] Worek F, Jenner J, Thiermann H (2016). Chemical warfare toxicology.

[CR94] Wrba L, Palmer A, Braun CK, Huber-Lang M (2017). Evaluation of gut-blood barrier dysfunction in various models of trauma, hemorrhagic shock, and burn injury. J Trauma Acute Care Surg.

[CR95] Wrba L, Ohmann JJ, Eisele P, Chakraborty S, Braumuller S, Braun CK, Klohs B, Schultze A, von Baum H, Palmer A, Huber-Lang M, Halbgebauer R (2019). Remote intestinal injury early after experimental polytrauma and hemorrhagic shock. Shock.

[CR96] Young, MK, Leslie, JL, Madden, GR, Lyerly, DM, Carman, RJ, Lyerly, MW, Stewart, DB, Abhyankar, MM, Petri, WA (2022) Binary toxin expression by Clostridioides difficile is associated with worse disease. Open Forum Infect Dis, 9(3). 10.1093/OFID/OFAC001 10.1093/ofid/ofac001 PMC8825761 35146046

[CR97] Zeiser J, Gerhard R, Just I, Pich A (2013). Substrate specificity of clostridial glucosylating toxins and their function on colonocytes analyzed by proteomics techniques. J Proteome Res.

[CR98] Zhu, Z, Schnell, L, Müller, B, Müller, M, Papatheodorou, P, Barth, H (2019) The Antibiotic bacitracin protects human intestinal epithelial cells and stem cell-derived intestinal organoids from Clostridium difficile toxin TcdB. Stem Cells Int, 2019. 10.1155/2019/4149762 10.1155/2019/4149762 PMC6701344 31467562

